# SPR Sensor Based on a Concave Photonic Crystal Fiber Structure with MoS_2_/Au Layers

**DOI:** 10.3390/ma16165523

**Published:** 2023-08-08

**Authors:** Xiaotong Guo, Yueke Wang, Tian Sang, Guofeng Yang, Qi Yao

**Affiliations:** 1 Optica Information Science and Technology Department, Jiangnan University, Wuxi 214122, China; 13766867579@163.com (X.G.); sangt@jiangnan.edu.cn (T.S.); gfyang@jiangnan.edu.cn (G.Y.); 2 China Optoelectronic Engineering and Technology Research Center, Jiangnan University, Wuxi 214122, China; 3 Zhejiang Beyondsun Green Energy Technology Co., Ltd., Huzhou 313008, China; 18851567612@163.com

**Keywords:** photonic crystal fiber, surface plasmon resonance, MoS_2_, wavelength sensitivity

## Abstract

We propose a surface plasmon resonance (SPR) sensor based on the concave photonic crystal fiber (PCF) coated with molybdenum disulfide (MoS_2_) and Au layers, which can detect the refractive index (RI) of the analyte. The finite element method (FEM) was used to verify our design, and the loss spectra of the fundamental mode are calculated. Compared with the SPR sensor with only a Au layer, the wavelength sensitivity can be improved by from 3700 to 4400 nm/RIU. Our proposed sensor works in near-infrared band and has a wide RI range from 1.19 to 1.40. The influences of the geometrical parameters of PCF and the thicknesses of Au and MoS_2_ layers on the loss spectra are discussed in detail, and the maximum wavelength sensitivity of 5100 nm/RIU can be achieved. Meanwhile, a high resolution of 1.96 × 10^−5^ RIU and the largest FOM of 29.143 can be obtained. It is believed that our findings show the sensor’s excellent potential in medical testing, unknown biological detection, environmental monitoring and organic chemical detection.

## 1. Introduction

Surface plasmon resonance (SPR) is widely studied due to its unique properties such as a short wavelength, near-field enhancement and good locality, as well as its being sensitive to the changes in the refractive index (RI) of analytes [1,2,3,4]. Therefore, SPR can be a candidate for physical, chemical and biological sensors [5,6], which have been used in many structures, including prism-coupled structures, optical waveguides, fibers and so on [7,8,9,10,11]. However, these configurations are too bulky and costly, as well as difficult to operate [12,13]. In 1993, Jorgenson proposed and demonstrated the first fiber-based SPR sensor [14], effectively addressing the limitations of conventional SPR sensors. Since then, there has been widespread research on a multitude of fiber-based SPR sensors. At present, photonic crystals are widely employed in various applications, including photonic crystal coupling emission platforms [15], and biochemical sensing [16,17]. SPR sensors based on photonic crystal fiber (PCF) attract more attention because of their controllable optical properties [18,19]. As is well-known, PCF-SPR sensors are usually based on the coupling between the fundamental mode of PCF and the surface plasmon polariton (SPP) mode. The SPP mode is a specific electromagnetic mode, which is a collective charge oscillation propagating along the interface between a metal and a dielectric material. The fundamental mode is the lowest mode in PCF, propagating along the core through total internal reflection. When phase-matching between SPP mode and the fundamental mode occurs, the SPR is excited and the loss of the fundamental mode reaches its maximum.

Sensor performance can be evaluated for sensitivity, RI detection range, and manufacturing cost [18,20,21,22,23,24,25]. At present, a variety of PCF-SPR sensor structures have been reported to improve sensor performance, which can be categorized into two classes [26]. For the first method, the sensing layer is coated inside the air holes of the PCF, and then the analyte is injected, which achieves a higher sensitivity [27]. However, this technique is difficult to fabricate, and the fiber mode propagates with large propagation loss. For the second method, the sensing layer is coated outside of the PCF, such as the polished D-type structure, and this fabrication can be easily achieved. In a further way, the microgroove structure of concave PCF can reduce the distance between the fiber core and analyte, which can promote the interaction between the fiber mode and SPP mode, enhancing the wavelength sensitivity. The selection of suitable plasmonic materials (sensing layer) is crucial, and Au is believed to be a good choice due to its superior chemical stability. Recently, two-dimensional transition metal dichalcogenides (TMDCs) such as molybdenum disulfide (MoS_2_) have been widely employed in sensing applications. Compared with graphene, MoS_2_ has a higher optical absorption efficiency due to its larger imaginary part of the dielectric constant, enhancing the excitation of SPR and the wavelength sensitivity [28,29,30,31,32,33].

In this paper, a novel PCF-SPR sensor coated with MoS_2_/gold layers in the near-infrared range has been proposed. The fundamental mode can be coupled with the SPP mode, which can be used to detect the RI of the analyte. The simulation results based on finite element method (FEM) show the wavelength sensitivities are 3700 and 4400 nm/RIU for the sensor with only gold layer and MoS_2_/gold layers, respectively. It is believed that the MoS_2_ layers enhance the sensing performances of our design. The loss spectra under various parameters, including the diameters and pitch of air holes, the radius of the microgroove, the distance between the microgroove and fiber core, and the thicknesses of Au and MoS_2_ layers, are discussed. Therefore, the wavelength sensitivity, resolution and FOM can reach 5100 nm/RIU, 1.96 × 10^−5^ RIU and 29.143, respectively. The proposed sensor with MoS_2_/gold layers would be a good choice for a wide range (from 1.19 to 1.40) of RI detection with high accuracy, which has potential in pharmaceutical inspection, environmental monitoring, and bio-sensing.

## 2. Structural Design of the Sensor

**Figure 1:**
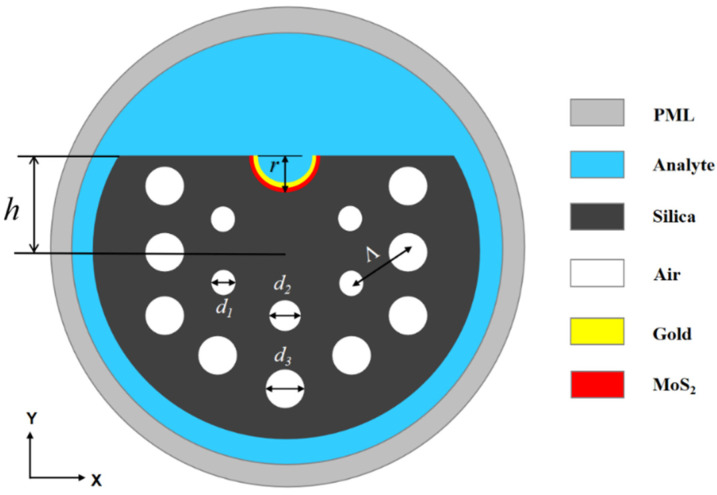
Cross-section of the PCF-SPR sensor.

The proposed sensor, based on a concave photonic crystal fiber with MoS_2_/gold layers, is shown in Figure 1. The fabrication of the structure involves the precision polishing of the upper section of the PCF, and the etching of a microgroove with radius *r* (=2 μm). The MoS_2_ layer is transferred to the top circular portion, and the gold layer is coated on the MoS_2_ [34,35,36,37,38]. Here, the distance between the microgroove and fiber core is *h* (=4 μm), and the thickness of the gold layer is *t* (=50 nm). To prevent energy leakage from the fiber core into the cladding, the structure consists of two hexagonal-ring air holes. The inner ring consists of two kinds of air holes, the diameters of which are *d*_1_ (=1.2 μm) and *d*_2_ (=1.5 μm), respectively. The air holes in the outer ring are fixed at the diameter *d*_3_ (=1.8 μm), and the pitch of the holes is *Λ* (=2 μm). In the process of preparing PCF, incorporating air holes with different sizes, the quartz capillaries are first stacked. Then, the quartz capillaries are drawn to achieve different sizes, and the drawing parameters can be controlled by inflation, evacuation and sealing methods [39].

Here, the background material of PCF is fused silica. The RI of the silica is calculated by the Sellmeier formula as Equation (1) [40].

(1) $$n_{silica}=\sqrt{1+\sum_{i=1}^{3}\frac{a_{i}\lambda^{2}}{\lambda^{2}-b_{i}^{2}}}$$

Here, *n_silica_* is the RI of silica, *λ* is the operating wavelength and *a*_1_ = 0.6961, *a*_2_ = 0.4079, *a*_3_ = 0.8974, *b*_1_ = 0.0683 μm, *b*_2_ = 0.1155 μm, and *b*_3_ = 9.8961 μm. The permittivity of gold can be represented by the Drude–Lorentz model as Equation (2) [41].

(2) $$\varepsilon =\varepsilon_{\infty }-\frac{\omega_{D}^{2}}{\omega (\omega +j\gamma_{D})}+\frac{\Delta \varepsilon \cdot \Omega_{L}^{2}}{(\Omega_{L}^{2}-\omega^{2})-j\Gamma_{L}\omega }$$

Here, *ε_∞_* (=5.9673) is the high-frequency dielectric constant of gold, *γ_D_* (=15.92 × 2π THz) is the damped frequency, *ω_D_* (=2113.6 × 2π THz) is the plasmon frequency, Δ*ε* (=1.09) is the quality factor, Г*_L_*(=104.86 × 2π THz) is the spectrum width, Ω*_L_*(=650.07 × 2π THz) is the frequency of the Lorentz oscillator, and *ω* is the angular frequency of incident light. The RI of the MoS_2_ can be found in Ref. [30]. For the proposed structure working as a sensor, a crucial requirement is the achievement of phase-matching between the fundamental mode and the SPP mode. When the phase-matching is satisfied, the SPR happens and the loss of the fundamental mode of fiber reaches its maximum. The propagation loss of the fiber mode can be calculated as Equation (3) [42].

(3) αloss(dB/cm)=8.686×2πλIm(neff)×104

Here, *λ* is the operating wavelength, Im(*n_eff_*) is the effective RI of the imaginary part, and *α_loss_* is proportional to the Im(*n_eff_*). When the RI of the analyte changes, so does the phase-matching condition, which leads to a change in the loss peak for the fundamental mode. Therefore, the SPR sensor based on PCF is achieved. In this paper, we use an RF module of COMSOL Multiphysics 6.0 to perform the finite element method (FEM) simulations. In addition, dense free triangle meshes and a perfectly matched layer (PML) of 3 μm are employed for accuracy. Around the simulation region, the perfect matching layer (PML) is introduced to absorb the outgoing electromagnetic wave.

## 3. Results and Discussions

**Figure 2:**
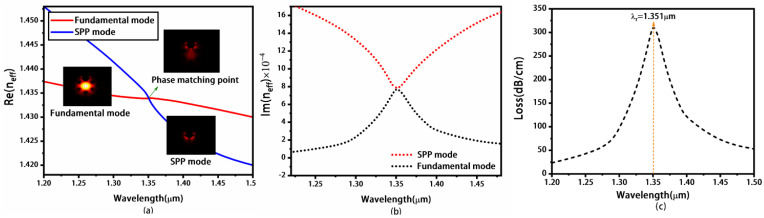
The real part (**a**) and imaginary part (**b**) of the effective refractive index for the y-polarization fundamental mode and the SPP mode, respectively. The corresponding loss spectrum of the y-polarization fundamental mode (**c**). Here, *n_a_* is chosen as 1.30 and the concave photonic crystal fiber is coated with only a gold layer with 50 nm thickness.

Firstly, we discuss the concave photonic crystal fiber with only a gold layer of 50 nm thickness (as shown in Figure 1 and without MoS_2_ layers). In our work, we only consider the y-polarization fundamental mode resulting in the excitation of more free surface electrons, which has a larger evanescent field than the x-polarization mode. Thus, the y-polarization mode is easily coupled with the SPP mode. Figure 2a shows the dispersion relations of the y-polarization fundamental and the SPP modes, and the RI of the analyte *n_a_* is 1.30. The red and blue curves represent the real part of *n_eff_* (Re(*n_eff_*)) of the fundamental and the SPP modes, respectively. The dispersions of the two modes both descend with increasing wavelength, and the descending speed of SPP mode is faster than that of the y-polarization fundamental mode. This is because that SPP mode has a larger dispersion compared with the y-polarization fundamental mode. Then, the Re(*n_eff_*) of both modes coincide with each other at *λ_r_* of 1.351 μm, which indicates that the SPP mode couples with the y-polarization fundamental mode. Thus, the phase-matching condition is satisfied, and the SPR phenomenon occurs. At the same time, the Im(*n_eff_*) of the two modes is equal at *λ_r_* of 1.351 μm, as shown in Figure 2b. Figure 2c shows the propagation loss spectrum of the fundamental mode based on Equation (3). There is an obvious peak in the loss spectra of the y-polarization fundamental mode, which reaches the maximum propagation loss of 313.318 dB/cm, corresponding to SPR.

**Figure 3:**
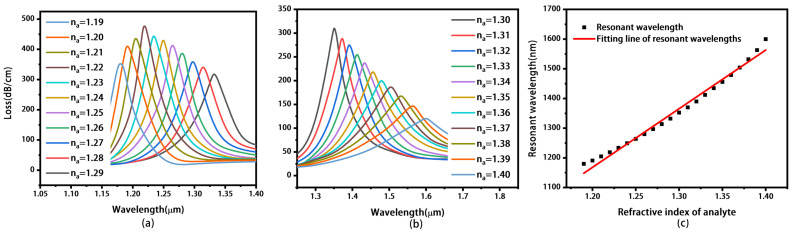
Loss spectra of the sensor with only gold coating when the analyte ranges from 1.19 to 1.29 (**a**), and from 1.30 to 1.40 (**b**). Fitting line of resonant wavelengths (**c**).

Figure 3a,b illustrate the loss spectra of the proposed sensor without MoS_2_ layers under different RI of analyte. When RI increases from 1.19 to 1.40, the loss peak moves to longer wavelength, increasing from 1180 to 1600 nm. When the RI of the analyte exceeds 1.40, the coupling between the fundamental mode and the SPP mode is so weak that it is not suitable for sensing. The values of the loss peaks first increase and reach a maximum of 480.127 dB/cm at *n_a_* = 1.22. Then, the value of the loss peak decreases to 122.972 dB/cm. Thus, the SPR sensor is achieved, and the RI range of the analyte is so wide that our design can be used for pharmaceutical inspection, leakage monitoring, and bio-sensing [43].

Figure 3c shows the fitting line of the resonant wavelength with RI increasing from 1.19 to 1.40. It can be seen that there is a good linear relationship between the RI of the analyte and the resonant wavelength. The wavelength sensitivity of the proposed sensor can be calculated as Equation (4) [44].

(4) $$S_{\lambda }(\mathrm{nm}/\mathrm{RIU})=\frac{\Delta \lambda_{peak}}{\Delta n_{a}}$$

Here, Δ*λ_peak_* is the resonant wavelength shift of the loss spectra, and Δ*n_a_* is the change in the RI of the analyte. The values for Δ*λ_peak_* and Δ*n_a_* can be obtained from Figure 3a,b. The maximum sensitivity of 3700 nm/RIU is obtained based on Equation (4), when *n_a_* increases from 1.39 to 1.4. The resolution *R* of the sensor can be calculated as Equation (5) [45].

(5) $$R(\mathrm{RIU})=\frac{\Delta n_{a}\times \Delta \lambda_{\min}}{\Delta \lambda_{peak}}$$

Here, Δ*λ_min_* is 0.1 nm, which is the minimum spectral resolution. A high resolution of 2.7 × 10^−5^ RIU is obtained when *n_a_* = 1.39 in the proposed structure. Figure of merit (FOM) serves as a parameter of sensor performance, which is defined as in Equation (6) [46,47].

(6) $$\mathrm{FOM}=\frac{S_{\lambda }}{\mathrm{FWHM}}$$

Here, FWHM is the full-width at half-maximum of the resonance wavelength peak. The corresponding FOM is 11.974 when *n_a_* = 1.39.

**Figure 4:**
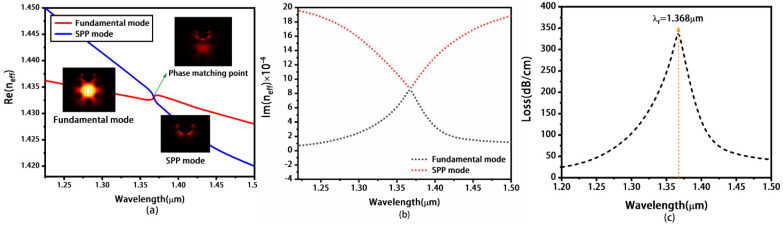
The real part (**a**) and imaginary part (**b**) of the effective refractive index for the y-polarization fundamental mode and the SPP mode, respectively. The corresponding loss spectrum of the y-polarization fundamental mode (**c**). Here, *n_a_* is chosen as 1.30 and the concave photonic crystal fiber is coated with both MoS_2_ and gold layers.

Secondly, we discuss the concave photonic crystal fiber with both MoS_2_ and gold layers (as shown in Figure 1), and other parameters are the same as those of Figure 2. The number of layers for MoS_2_ was chosen to be two and the thickness of monolayer is 0.65 nm. Figure 4a shows the dispersion relations of the y-polarization fundamental (denoted by red line) and the SPP mode (denoted by blue line). Similar to the results of Figure 2a, when the wavelength increases from 1.20 to 1.50 μm, the real parts of the two modes decrease. The descending speed of the SPP mode is faster than that of the y-polarization fundamental mode, and the Re(*n_eff_*) of the two modes coincides at *λ_r_* of 1.368 μm, which demonstrates that the SPP mode couples with the y-polarization fundamental mode and SPR occurs. Figure 4b represents the Im(*n_eff_*) of the two modes, and the value of Im(*n_eff_*) of SPP mode is equal to that of the y-polarization fundamental mode at the resonant wavelength *λ_r_* of 1.368 μm. Figure 4c presents the loss spectra, revealing the emergence of a distinct peak with a maximum propagation loss of 339.978 dB/cm.

**Figure 5:**
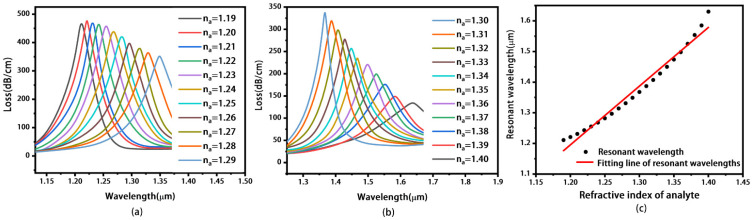
Loss spectra of the sensor with both MoS_2_ and gold coating when the analyte ranges from 1.19 to 1.29 (**a**), and from 1.30 to 1.40 (**b**). Fitting line of resonant wavelengths (**c**).

Figure 5a,b illustrate the loss spectra of the proposed sensor with both MoS_2_ and gold coatings under different RIs. We can see that with the increase in the RI from 1.19 to 1.40, the loss peak moves from 1211 to 1630 nm. Thus, the SPR sensor is realized. The value of the resonance peak first increases and then decreases, and reaches a maximum of 478.915 dB/cm at *n_a_* = 1.20. The maximum sensitivity of 4400 nm/RIU is obtained when *n_a_* increases from 1.39 to 1.40, which is larger than that of Figure 3. The corresponding resolution is 2.22 × 10^−5^ RIU and FOM is 25.143 when *n_a_* = 1.39. Compared with the results of Figure 3, the structure with MoS_2_ layers has a better sensor performance. Figure 5c shows the fitting line of resonant wavelength under different RI. Our findings demonstrate a linear correlation between the refractive index (RI) of the analyte and the resonant wavelength. This good linearity enhances measurement accuracy and reliability, thereby validating the rationality of our sensor design [48].

**Figure 6:**
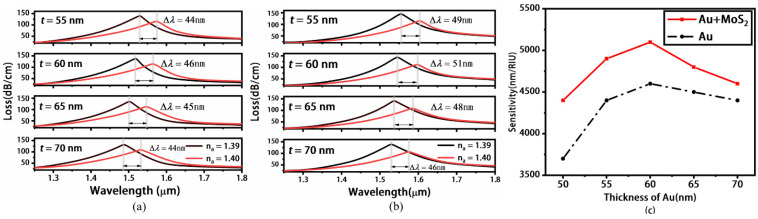
Loss spectra under different gold thickness *t* when the concave photonic crystal fiber is coated with gold layers (**a**) and gold/MoS_2_ layers (**b**), respectively. The corresponding wavelength sensitivity under different gold thickness *t* (**c**). Here, *n_a_* is chosen as 1.39 and 1.40.

As is well-known, the thickness of the metal layer has a significant influence on the sensing performance of SPR sensors. We calculate the sensitivity under different thickness *t* of Au layer, as shown in Figure 6, and form a comparison between sensors with MoS_2_ and without MoS_2_. In our design, the maximum sensitivity is determined by Δ*n_a_* when *n_a_* increases from 1.39 to 1.40. Thus, we only show the loss spectra when *n_a_* is 1.39 and 1.40, and calculate the sensitivity under different thicknesses of *t*. Figure 6a shows the loss spectra when *t* = 55, 60, 65, 70 nm when RI increases from 1.39 to 1.40 without MoS_2_ layers. The shifts in Δ*λ_peak_* of the loss peaks are 44, 46, 45 and 44 nm, respectively. Figure 6b shows the loss spectra for the sensor with MoS_2_ when RI increases from 1.39 to 1.40; the resonance peak shifts are 49, 51, 48 and 46 nm for *t* = 55, 60, 65 and 70 nm, respectively. The sensitivity of sensors with MoS_2_ and without MoS_2_ layers is shown in Figure 6c. The red(black) curve shows that the sensitivity of a sensor with MoS_2_(without MoS_2_) increases from 4400(3700) to 5100 nm/RIU (4600 nm/RIU) when *t* increases from 50 to 60 nm, and then decreases to 4600 nm/RIU (4400 nm/RIU) from 60 to 70 nm. Obviously, the resonance peak shifts in the sensor with MoS_2_ are always larger than those of the sensor without MoS_2_ under different a *t*, as are the shifts in sensitivity. When *t* is chosen as 60 nm for the sensors with MoS_2_, the sensitivity (=5100 nm/RIU) reaches the maximum.

**Figure 7:**
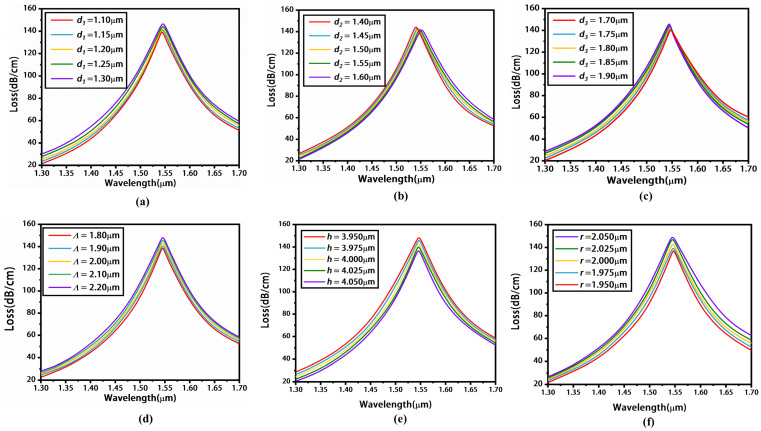
Loss spectra under different parameters *d*_1_ (**a**), *d*_2_ (**b**), *d*_3_ (**c**), *Λ* (**d**), *h* (**e**), and *r* (**f**). Here, *n_a_* is chosen as 1.39.

Next, we discuss the influence of parameters, including air hole diameters *d*_1_, *d*_2_, *d*_3_, and the pitch *Λ* of the holes, on the loss spectra of sensor with MoS_2_. Here, the thickness of the Au layer was chosen to be 60 nm, and other parameters were the same as those in Figure 5. When *d*_1_ varies from 1.10 to 1.30 μm, the loss peak values show a small increase and the position of the resonant wavelength remains almost unchanged, as shown in Figure 7a. Figure 7b shows the variation in loss spectra with *d*_2_ changing from 1.40 to 1.60 μm. The value of the loss peak decreases slightly, and the resonant wavelength shifts from 1.540 to 1.552 μm. Figure 7c shows the variation in the loss spectra with *d*_3_ from 1.70 to 1.90 μm. The result shows that, with the decrease in *d*_3_, the value of the resonance peak has a minor increase and the resonant wavelength redshifts from 1.544 μm to 1.547 μm. Figure 7d shows the variation in loss spectra with *Λ* changing from 1.80 to 2.20 μm. The resonant wavelength is unchanged with *Λ*, and a higher pitch value enhances the coupling between fundamental mode and SPP mode, leading to larger loss. Figure 7e shows the variation in loss spectra with *h* changing from 3.95 to 4.05 μm. With increasing *h*, the resonance peak remains consistent at a wavelength of 1546 nm, and the loss peak value decreases. This is because a smaller *h* leads to stronger coupling between SPP mode and y-polarization fundamental mode. As shown in Figure 7f, when *r* ranges from 1.95 to 2.05 μm, the loss peak experiences a slight increment while the resonant wavelength remains nearly constant. Considering the medium value of the loss peak, we finally selected the optimal diameters of *d*_1_ (=1.2 μm), *d*_2_ (=1.5 μm), *d*_3_ (=1.8 μm), *Λ* (=2 μm), *h* (=4 μm), and *r* (=2 μm).

**Figure 8:**
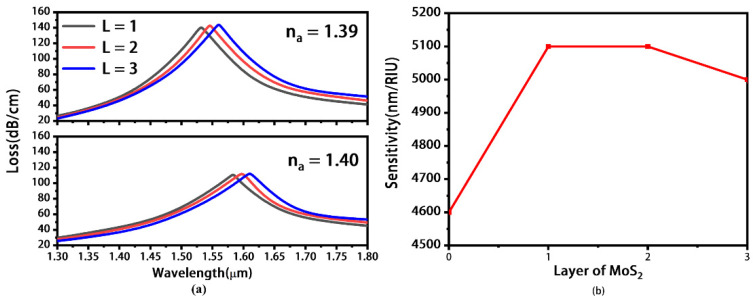
Loss spectra under different numbers of MoS_2_ layers (**a**) and the corresponding wavelength sensitivity (**b**). Here, *n_a_* is chosen as 1.39 and 1.40.

Figure 8 shows the variation in the maximum wavelength sensitivity under different numbers of MoS_2_ layers. The thickness of Au layer is 60 nm, and other parameters are the same as those in Figure 5. Figure 8a shows the loss spectra with the number of MoS_2_ layers increasing from 0 to 3. It is found that the resonance wavelength shifts from 1.532(1.583) to 1.560 μm (1.610 μm) when *n_a_* = 1.39(1.40). When the number of MoS_2_ layers is larger than 3, the coupling between SPP and fundamental mode is so weak that the loss peak cannot be found. Figure 8b shows the maximum sensitivity under different numbers of MoS_2_ layers, and the sensitivity reaches the peak value of 5100 nm/RIU when L = 1 and 2. Finally we selected double layers of MoS_2_ due to its high sensitivity and sharp resonance peak. Table 1 shows the performances of similar SPR sensors in the communication region. Compared with those works, our sensor has a wider and better wavelength sensitivity.

## 4. Conclusions

In this paper, we propose a novel concave PCF-SPR sensor with a two-dimensional material, MoS_2_, which works in near-infrared range. The concave structure can reduce the distance between the fiber core and the analyte, which is more efficient for the coupling between SPP and the fundamental modes. The wavelength sensitivity of the sensor with a gold/MoS_2_ coating can be improved by 700 nm/RIU, compared with that with only a gold layer. By discussing the influences of various parameters on the loss spectra in detail, the maximum wavelength sensitivity (=5100 nm/RIU), a high resolution (=1.96 × 10^−5^ RIU) and an FOM of 29.143, can be achieved. Therefore, our design could be a good candidate for unknown biological detection, environmental monitoring and pharmaceutical inspection.

## Figures and Tables

**Table 1:** Comparison of the proposed sensor with recently reported SPR sensors.

Ref	Structure Configuration	S_λ_ (nm/RIU)	R (RIU)	RI Range	Working Wavelength Range (nm)	FOM
[49]	Al/zinc oxide microchannel PCF-SPR sensor	5000	2.00 × 10^−5^	1.32–1.34	1805–1885	/
[33]	Gold/graphene coated concave-shaped PCF-SPR sensor	2290	/	1.33–1.3688	600–690	/
[50]	Ag/Graphene coated D-shaped PCF-SPR sensor	3700	4.60 × 10^−5^	1.33–1.37	500–610	/
[51]	Gold grating D-shaped PCF-SPR sensor	3340	5.98 × 10^−6^	1.36–1.38	1550–1625	33.4
[52]	Gold coated hollow core D-shaped PCF-SPR sensor	2900	/	/	/	/
[53]	Gold coated PCF-SPR sensor	4156	2.41 × 10^−5^	1.29–1.49	1050–1700	/
Proposed	MoS2/gold coated concave-shaped PCF-SPR sensor	5100	1.96 × 10^−5^	1.19–1.40	1211–1630	29.143

## Data Availability

Data will be made available on request.
